# Effective operating room (OR) utilization by performing low-complex surgical procedures during the 2020 corona pandemic

**DOI:** 10.1007/s11548-021-02392-3

**Published:** 2021-05-17

**Authors:** Thomas Vogel, Dina Schippers, Balqees Aldarweesh, Ilaria Pergolini, Martina Stollreiter, Klaus Wagner, Dirk Wilhelm, Helmut Friess, Michael Kranzfelder

**Affiliations:** 1 Department of Surgery, Klinikum rechts der Isar, Technische Universität München, Ismaninger Strasse 22, 81675 Munich, Germany; 2 Department of Anaesthesiology, Klinikum rechts der Isar, Technische Universität, Munich, Germany; 3 Klinikum rechts der Isar, Zentrum Ambulante Chirurgie (ZAC), Technische Universität, Munich, Germany

**Keywords:** COVID-19 pandemic, Low-complex surgery, Central-venous port catheter, Effective OR utilization, Surgical workflow optimization

## Abstract

**Purpose:**

The SARS-CoV-2 pandemic has almost stopped all elective surgical treatment throughout the world. As operating room (OR) capacities are reduced everywhere to ensure availability of intensive care capacities, especially low-complex surgical procedures are often postponed. These include totally implantable central-venous access ports which are important for the oncologic treatment of cancer patients.

**Methods:**

In our study, we investigated the potential of an outpatient surgical centre (OSC) in terms of workflow effectiveness compared to the central operating room complex (COR) of a university hospital using low-complex surgical procedures as an example. Data of 524 consecutive patients who received a Port-a-cath procedure (422 implantations (80.5%) and 102 explantations (19.5%)) in our department between February 2019 and February 2020 were evaluated.

**Results:**

A total of 277 patients were operated in outpatient surgical centre (OSC), and 247 patients received the procedure in the central OR (COR) complex. Grade II and III complications according to the Clavien–Dindo classification occurred in 5.2% (OSC) and 7.3% (COR) of patients. Incision-to-suture time was significantly quicker in the OSC group (36 vs. 42 min., *p* < 0.032). Total OR time (01:08 vs. 01:20 h) and preparation-to-incision time were also shorter in the OSC group (12 vs. 17 min., *p *< 0.002).

**Conclusion:**

In order to ensure effective OR utilization especially in times of the corona pandemic, the use of smaller decentralized OR units, e.g., outpatient surgical centres, for performing low-complex surgical cases is beneficial. Our study revealed shorter total OR and preparation-to-incision times.

## Introduction

As a result of hospital capacity constraints and the swift recognition that COVID‐19 poses an important danger to both patients and healthcare professionals [1], the SARS-CoV-2 pandemic has almost stopped all elective surgical treatment throughout the world [2]. Operating room (OR) capacities are reduced everywhere to ensure availability of intensive care capacities. Emergency and cancer operations are still performed; however, OR capacity for low-complex surgical procedures is very limited. These include totally implantable central-venous access ports (Port-a-cath) which are urgently needed for the oncologic treatment of cancer patients [3]. Without a prompt start of chemotherapy, patients' chances of survival decrease.

Compared to the central OR (COR) complex of a hospital, where workflows are expected to be slower due to the broader range of procedures, length of operations and scope of organization [4], low-complex surgical procedures (such as Port-a-cath procedures) can possibly be performed more effectively in an outpatient surgical centre (OSC). Thereby, OR time and capacity in the central OR complex can be saved and used, e.g., for emergency cases [5].

## Materials and method

In this study, we evaluated data of 524 consecutive patients who received a Port-a-cath procedure in the Department of Surgery, Klinikum rechts der Isar, Technical University Munich (MRI) between February 2019 and February 2020, whereas 277 patients were operated in the outpatient surgery centre (OSC), 244 patients received the procedure in the central OR (COR) complex. Patient assignment to the OSC and COR was done randomly. However, urgent port implantations or explantations during on-call hours were performed in the central operating room. All operations were performed under local anesthesia [6]. Scheduling of the patients OR slot was done by our central patient management using SAP/KIS software. All patients were seen for follow-up 7–10 days after surgery in the outpatient clinic, patients with Port-a-cath implantation were also seen 3 months postoperatively (first tumor follow-up). After beginning of the COVID-19 pandemic, all patients had a negative SARS-CoV-2 PCR test result prior to surgery. Besides descriptive statistics of the study cohort, incision-to-suture time, total OR time and preparation-to-incision time were analyzed. Patient satisfaction was assessed by using a self‐administered questionnaire in the OSC cohort. Statistical analysis was performed using Microsoft© Excel 2016 and IBM© SPSS statistics 27.

## Results

A total of 422 patients (80.5%) received a Port-a-cath implantation (BARD Access Systems, Inc., Salt Lake City, USA) due to cancer diagnosis for chemotherapy. In 102 patients (19.5%), the Port-a-cath was removed after chemotherapy was completed or due to complications (port thrombosis, infection, malfunction). For port implantation, cut down technique of the cephalic vein (open strategy) was performed in *n* = 359 patients (85.1%). In *n* = 63 patients (14.9%), puncture of the subclavian vein (closed strategy) was necessary. No grade IV or V complication according to the Clavien–Dindo classification occurred, especially no pneumo- or hematothorax after vein puncture or postoperative bleeding with the need of surgical revision was observed.

In the OSC cohort, 229 patients (82.7%) received a Port-a-cath implantation and 48 patients (17.3%) a Port-a-cath explantation.

Postoperative complications grade II and III according to the Clavien–Dindo classification occurred in 12 patients with Port-a-cath implantation (5.2%). Of these, 5 (2.2%) were perioperative (inadvertent arterial puncture) and 7 (3.1%) were long-term complications (infection and thrombosis).

In the COR cohort, 193 patients (78.1%) received a Port-a-cath implantation and 54 patients (21.9%) a Port-a-cath explantation. Postoperative complications grade II and III occurred in 14 patients with Port-a-cath implantation (7.3%). Of these, 4 (2.1%) were perioperative (inadvertent arterial puncture) and 10 (5.2%) were long-term complications (infection and thrombosis).

Table 1 summarizes the results of incision-to-suture time, total OR time and preparation-to-incision time. Preprocessing covers the period between the patient's call to the OR and the end of positioning on the OR table, postprocessing comprises the period after completion of the patient`s procedure and leaving the OR.

Intraoperative fluoroscopy images (Ziehm Vision FD, Ziehm Imaging GmbH, Nürnberg Germany) were transferred to the Picture Archiving and Communication System (PACS) via LAN interface using the SAP/KIS compatible worklist of the C-arm software. Conventional postoperative X-ray for exclusion of pneumothorax was performed if the subclavian vein (closed strategy) was punctured.

Patient satisfaction was assessed by using a self‐administered questionnaire in the OSC cohort. The scheduling of the surgery date was considered very good by 74% and good by 22.4% of patients. Scheduling of the time slot on the respective OR date was considered very good by 69.6% and good by 25.6% of patients. 3.9% were not satisfied (waiting time to long). The overall patient management was rated very good by 79.8% and good by 3.9% of patients; however, 15.6% did not answer this question. Recommendation for others to have an operation performed in the OSC was made by 75.9% of patients.

## Conclusion

Primary success rates, tolerability, grade II and III complication rates according to the Clavien–Dindo classification and dose rate of radiation did not differ significantly between the groups. No grade IV and V complication was noted. All patients were seen for follow-up 7–10 days after surgery in the outpatient clinic, patients with Port-a-cath implantation were also seen 3 months postoperatively (first tumor follow-up).

Patient assignment to the OSC and COR was done randomly. However, urgent port implantations or explantations during on-call hours were performed in the central operating room. Mean incision-to-suture time for Port-a-cath explantation was similar between the OSC and COR cohort, however, it was significantly quicker in the OSC group (36 vs. 42 min., *p* < 0.032). This could be due to the fact, that the procedure was always performed or assisted by the same experienced consultant surgeon in the OSC group, whereas in the COR cohort, different surgeons (varied experience level) performed the procedure.

Total OR time, including pre- and postprocessing of the patient, was quicker in the OSC group (01:08 vs. 01:20 h) although the results did not differ significantly between the groups. However, the preparation-to-incision time differed significantly (12 min. (OSC) vs. 17 min. (COR), *p* < 0.002). The time savings can be explained by the fact that the medical and nursing staff in the outpatient surgical centre are better coordinated, communication structures are optimized and the distances in the OSC are generally shorter. The authors assume, that these time savings can be used as best practice for any hospital institution with identical OR structures. Scheduling of the OR date and time slot, as well as rating of the general patient management, was considered very good by 70–80% of patients.

In order to save OR capacity especially in times of the corona pandemic, were predominantly emergency cases and cancer operations should be performed in the central ORs of the hospitals, the use of smaller OR units, such as outpatient surgical centres, for low-complex surgical cases is beneficial. Our study revealed shorter total OR times and a quicker pre- and postprocessing (preparation-to-incision time), enabling a more effective OR utilization with the possibility to perform an increased number of low-complex operations without endangering patient safety.

**Table 1:** Incision-to-suture time, total OR time and preparation-to-incision time (Mean ± SD (h))

Incision-to-suture time	OSC		COR		*p*
	Mean (h)	± SD (h)	Mean (h)	± SD (h)	
Port-a-cath implantation	0:36	0:16	0:42	0:19	**0.032**
Port-a-cath explantation	0:26	0:12	0:27	0:16	0.379
Total OR time	1:08	1:03	1:20	0:25	0.264
Preparation-to-incision time	0:12	0:08	0:17	0:10	**0.002**

*OSC* outpatient surgical centre, *COR* central OR complex

Significance level *p* < 0.05

## Data Availability

Data supports their published claims and comply with field standards.
